# Electrochemical Oxidation of UV Filters: A First‐Principles Molecular Dynamics Study

**DOI:** 10.1002/chem.202402924

**Published:** 2024-11-16

**Authors:** Luis Álvarez, Irmgard Frank

**Affiliations:** ^1^ Institut für Physikalische Chemie und Elektrochemie Leibniz Universität Hannover Callinstr. 3A 30167 Hannover Germany

**Keywords:** *ab initio* molecular dynamics, Car-Parrinello molecular dynamics, ultraviolet filters, wastewater treatment, electrochemistry

## Abstract

A theoretical model is proposed to study the oxidation mechanisms of the organic UV filters **BP3** and **BP4** during electrochemical water treatment utilizing Car‐Parrinello molecular dynamics. Factors such as the amount of solvent to be included and how to design the system with the least possible intervention are discussed. The proposed model consist of the optimization of the geometries by density functional theory methods, the equilibration of the structure immersed in a water box, the inclusion of the reactive species, and the analysis of the reaction energies of each reaction pathway. The ab‐initio molecular dynamics simulations lead to several products, and some trends can be identified, in accordance with the well‐known reactivity rules of organic chemistry. The products proposed in this work are intermediates in longer oxidative pathways.

## Graphical abstract

In an in silico study, the reactions of water contaminated with UV filters in an oxidizing environment are simulated. By following the motion of Wannier centers, the movement of the orbitals during the reactions are tracked, thus distinguishing, for instance, redox reactions from acid‐base reactions. The reactivity trends and pathways are highlighted.

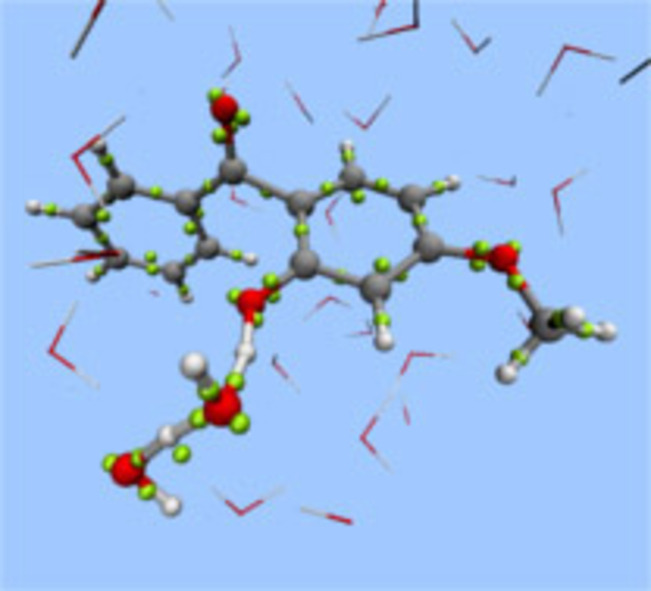

## Introduction

Over the past decades, the demand for sunscreens has increased due to a growing awareness of the risks associated with prolonged sun exposure. Currently, approximately 40 active ingredients have been registered in different countries, but only nine are widely employed. These include the inorganic compounds titanium dioxide (TiO_2_) and zinc oxide (ZnO), which are used as sunscreens, and seven organic UV filters: BMD, BP3, BP4, HS, ODP, OMS and OS.^[1]^ These organic compounds possess conjugated systems that allow them to absorb UV light, as well as hydrophilic and hydrophobic zones that permit their incorporation into the emulsion. In recent years, the environmental concentration of these compounds has increased, particularly in tourist zones, prompting the interest in the environmental impact of these compounds. It was found that the current concentrations of UV filters are reaching levels that can produce adverse effects, particularly on corals and microalgae.^[2]^ For these reasons, UV filters are considered as emerging contaminants, and there has been a growing interest in the development of wastewater treatment methods (**WWTMs**) that remove these compounds. The most common WWTM is chlorination, which has been linked to the formation of toxic by‐products, including trihalomethanes, haloacetic acid, and chloramines, among others. Consequently, alternative and supplementary methods are being investigated, including the incorporation of hydrogen peroxide and ozone as oxidizing agents.^[3]^ An additional alternative is electrochemical water treatment, since it provides a versatile, efficient, cost‐effective, easily automatable, and clean process.[^4^, ^5^] However, it is important to know what kind of products can be obtained when treating contaminated water with electrochemical methods, so it is necessary to study the possible oxidation mechanisms of these UV filters.

Computational chemistry is a link between theoretical and experimental chemistry. Through theoretical methods, experimental issues can be simulated, thereby facilitating the prediction of relevant results and properties of a system without spending significant time and resources and avoiding the production of chemical waste. Thus, the solution to industrial and environmental problems begins with the extrapolation of an in‐silico research. Car‐Parrinello Molecular Dynamics (**CPMD**)^[6]^ can be employed to determine reaction mechanisms on an ab‐initio level of theory. In this type of dynamics, nuclear motion is treated classically, while electrons are described by density functional theory (**DFT**). In this type of dynamics, the equations of motion for the nuclei and the electrons are solved simultaneously, considering that the electrons move in response to the forces generated by the nuclei and vice versa.

In CPMD, an extended Lagrangian (Eq. (1)) is introduced and solved using the Euler‐Lagrange equations (Eqs. (2) and (3)), thereby obtaining the Car‐Parrinello equations of motion for nuclei and electrons (Eqs. (4) and (5)). Hereby, M_*I*_ and R_*I*_ are the ionic masses and coordinates, *μ* is the fictitious electron mass, the $\psi_{i}$ are the orbitals, $E_{QC}=E^{KS}\left(\psi_{i},R\right)+V_{II}\left(R\right)$ is the potential energy, composed of the Kohn‐Sham energy and the nuclear‐nuclear potential energy, $\Lambda_{ij}$ is the Hermitian Lagrangian multiplier matrix, which introduces the orthogonality constraints of the system and $\upsilon_{eff}\left(r\right)$ is the effective potential in Kohn‐Sham theory. The addition of the term with the so‐called fictitious electron mass, *μ*, gives the orbital an artificial inertia, leading to a stable molecular dynamics in the picosecond range, which is more computationally economical than, for instance, Born‐Oppenheimer molecular dynamics (**BOMD**),^[7]^ where the Schrödinger equation must be solved iteratively for each step.

(1) $$ℒ=\sum_{I}\frac{1}{2}M_{I}{\dot{R}}_{I}^{2}+\sum_{I}\mu \int {\dot{\psi }}_{i}^{*}{\dot{\psi }}_{i}d\tau -E_{QC}\;\;+\sum_{i}\sum_{j}\Lambda_{ij}\left(\int \psi_{i}^{*}\psi_{j}d\tau -\delta_{ij}\right)$$

(2) $$\frac{d}{dt}\frac{\partial ℒ}{\partial {\underline{\dot{R}}}_{I}}=\frac{\partial ℒ}{\partial {\underline{R}}_{I}}$$

(3) $$\frac{d}{dt}\frac{\partial ℒ}{\partial {\dot{\psi }}_{i}^{*}}=\frac{\partial ℒ}{\partial \psi_{i}^{*}}$$

(4) $$M_{I}{\underline{\ddot{R}}}_{I}=-\frac{\partial }{\partial {\underline{R}}_{I}}E_{QC}$$

(5) $$\mu {\ddot{\psi }}_{i}=-\left[-\frac{1}{2}\nabla^{2}+\upsilon_{eff}\left(r\right)\right]\psi_{i}+\sum_{j}\Lambda_{ij}\psi_{j}$$

The objective of this research is to propose a theoretical model of the electrochemical treatment of water containing organic UV filters as pollutants using CPMD. The model is expected to predict their mechanisms of oxidation, and the products of those reactions. Benzophenone 3 (2‐hydroxy‐4‐methoxybenzophenone, **BP3**) and benzophenone 4 (5‐benzoyl‐4‐hydroxy‐2‐methoxybenzenesulfonic acid, **BP4**) were chosen for this study due to their small size and structural similarities.

To model an electrochemical oxidation, we introduce the organic UV filters in a suitable water box, and then we replace eight arbitrary water molecules with eight OH radicals. The resulting situation is highly reactive, and we observe spontaneous reactions in an unconstrained dynamics. The description of chemical reactions by unconstrained CPMD simulations has a long tradition[^7^, ^8^] and was performed before by several groups, see for example.[^9^, ^10^, ^11^] Constrained dynamics for the description of electrochemical reactions was performed using BOMD.[^12^, ^13^, ^14^]

## Results and Discussion

### Benzophenone‐3

**Figure 1:**
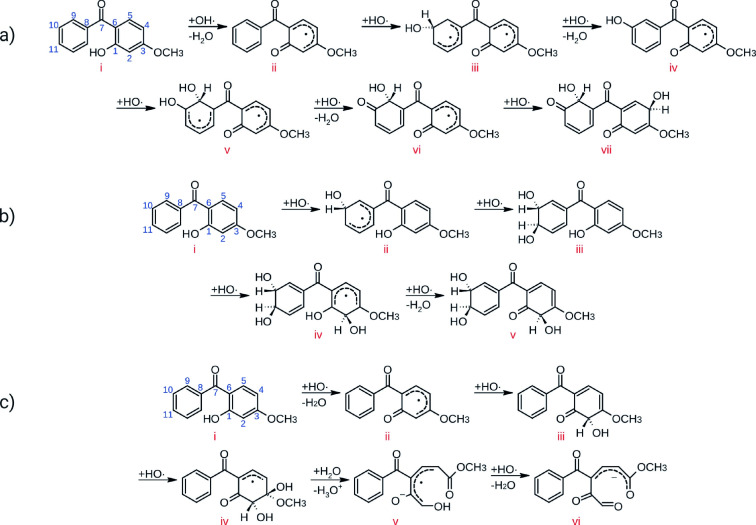
Selected mechanisms of oxidation of BP3. a) CPMD, NVE ensemble, density of the cell of 0.58 g cm^−1^; b) BOMD, NVE ensemble, density of the cell of 0.58 g cm^−1^; c) CPMD, NVT ensemble, density of the cell of 0.87 g cm^−1^.

The first molecule studied was **BP3**. When preparing the system, multiple factors were considered, including the type of dynamics (CPMD or BOMD) and the use of thermostats to control the temperature. In addition, the number of water molecules in the cell was varied. Several reaction mechanisms were observed, for a complete overview see Figure S1. We are highlighting those shown in Figure 1.

**Figure 2:**
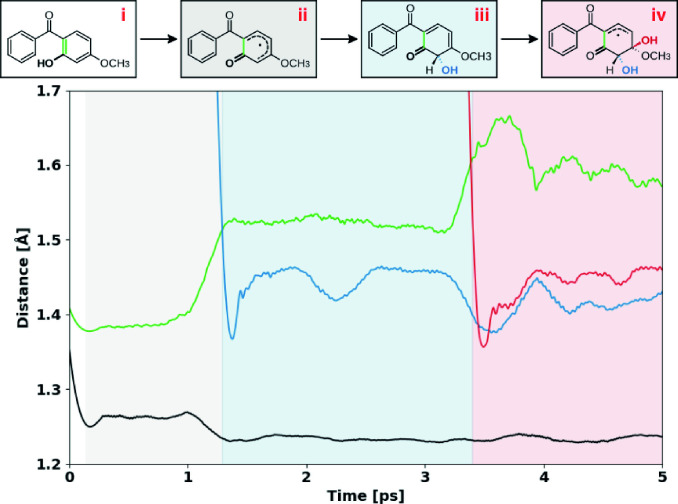
Evolution of interatomic distances between reactive species of reaction Figure 1c. Each line corresponds to the bonds highlighted by color in the structures.

The radical abstraction of phenolic hydrogen was observed in all routes, with this being the initial step in 80 % of the dynamics, as illustrated in Figure 1, mechanisms **a** and **c**. In mechanism **a**, the oxidation of the phenyl was observed, with the ketone moiety acting as a meta directing group, then, a second hydroxyl group was introduced at carbon 10, followed by the oxidation of carbon 9 (steps **iii** to **vi**). Finally, a last hydroxyl group was added to carbon 4, in ortho position to the former phenol moiety. In the phenyl ring, all reactions were addition/elimination, whereas in the phenol moiety, an elimination/addition mechanism was observed. In mechanism **b**, hydroxyl groups are added at carbons 10 and 11 respectively (steps **i** to **iii**). As these additions occur within less than 60 fs, they are considered as concerted reactions.^[15]^ Finally, a hydroxyl group is added to carbon 2, in ortho position to the phenol ring, and then, the phenol OH group at carbon 1 is oxidized to carbonyl, (steps **iii** to **v**). In mechanism **c**, following the abstraction of the phenolic hydrogen, the addition of hydroxyl groups at carbons 2 and 3 was observed (steps **ii** to **iv**). This intermediate reacted with water, forming a radical anion (step **iv** to **v**). Finally, the last hydroxyl group reacted, resulting in a highly oxidized product with four carbonyl groups (step **v** to **vi**). Steps **i** to **iii** of this mechanism were also observed in other dynamics runs, performed with different parameters (NVE ensemble, density of 0.58 g cm^−1^). Structural information, such as bond formation, bond cleavage, and changes in bond order, was obtained from the analysis of the atomic trajectories. For instance, in Figure 2 the relevant distances of the first steps in Figure 1c are shown. Furthermore, Wannier centers, which are centers of charge of localized orbitals,[^16^, ^17^] were computed. Thus, the position of these centers is associated with the charge distribution in the system. In case Figure 1c, the presence of unpaired electrons and the negative formal charge in the product was confirmed. With this information, we corroborated the proposed mechanisms.

The mechanisms described above can be summarized in the following stages: Firstly, the most acidic hydrogen is abstracted, in this case, the phenolic proton. Secondly, additions and aromatic substitutions occur in accordance with the ortho‐para director capacity of the phenol and the meta director capacity of the acyl group. Thirdly, the functional groups can undergo subsequent oxidation reactions. The ring closures and cleavages are conditioned to the position of the substituents (see the complete scheme in Figure S1).

In Table 1 the calculated reaction energies obtained using different DFT methods are shown as obtained in Gaussian calculations. Gaussian allows the comparison of the B3LYP and B2PLYP functionals. The B2PLYP functional deviates quite strongly from the corresponding BLYP and B3LYP values. Closer investigation reveals that this is due to a too low stability of the hydroxyl radical. The reasons of this deviation of the B2PLYP functional remain unclear, as our investigation shows that both B3LYP and MP2 are in close agreement concerning the stability of the hydroxyl radical.

**Table 1:** Reaction energies in kJ mol^−1^ of the selected reactions. For the detailed evolution of the energy during each step of these mechanisms, see Figure S2.

Reaction	**BLYP**	**BLYP** ^[a]^	**B3LYP**	**B3LYP** ^[a]^	**B2PLYP**
a) BP3+6 OH→3 H_2_O+**a.vii**	−1260.1	−1285.5	−1205.7	−1226.6	−978.9
b) BP3+4 OH→H_2_O+**b.v**	−760.5	−789.6	−736.0	−760.3	−588.8
c) BP3+3 OH→H_2_O+**c.iv** ^[b]^	−586.8	−611.5	−570.0	−590.8	−479.9

[a] Energies calculated employing empirical dispersion=gd3bj.^[21]^ [b] The energies correspond to the difference between steps **iv** and **i**, previous to the formation of the anion.

The additional Gaussian calculations are gas phase calculations. Hence, charged systems are not described correctly. In the CPMD calculations, ions are stabilized by the aqueous environment. This stabilization is problematic to describe at a pure ab‐initio level in Gaussian calculations, hence the ionic species, as formed in the route shown in Figure 1c, are not included in this comparison.^[18]^ The mechanisms obtained from the simulations in addition with the calculated enthalpies are in line with the reactivity trends observed in organic chemistry,[^19^, ^20^] thereby corroborating the conclusion that all the dynamics are consistent with what could be expected in a laboratory experiment. For example, we observe an ortho attack of the OH radical in structure iv of mechanism a) and a para attack of the OH radical in structure vi of mechanism a) which may ultimately lead to quinone formation.

### Benzophenone‐4

**Figure 3:**
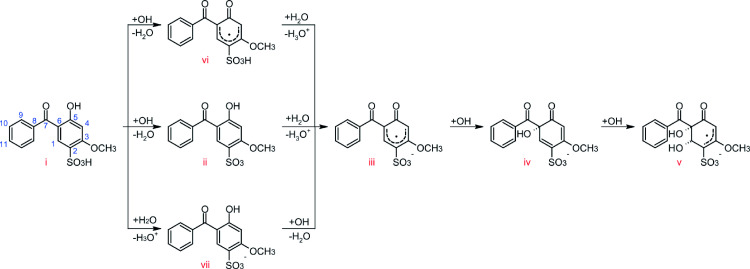
Oxidation mechanisms of BP4.

For this system, the comparison between NVE and NVT ensembles and different amounts of water were repeated, but no BOMD simulations were performed, as similar mechanisms were obtained with CPMD at a lower computational cost. In Figure 3, the oxidation pathways that **BP4** undergoes are shown. Structure **iii** was obtained as the final product in 58 % of the dynamics, while in 17 % of the dynamics it was obtained as an intermediate. Structure **iii** was obtained via three different routes, with route **i–vii‐iii** being the most common. This route consists in the abstraction of the proton from the sulfonic acid by water, followed by the abstraction of the phenolic hydrogen by a radical. Due to the acidic character of this compound, the Wannier centers were computed for each case, confirming the radical anion nature of structure **iii**, and that regardless of which of the three previous routes is followed, its electronic distribution is always the same, preferentially locating the radical electron at carbon 2.

The acid‐base reaction **i**–**vii** was even observed during the equilibration with water, prior to the simulation of the anodic environment. Some dynamics runs also exhibited a chemical equilibrium of the form R‐SO_3_H+H_2_O$\overset{⇀}{↽}$ R‐SO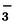 +H_3_O^+^ and R‐SO_3_H+OH$\overset{⇀}{↽}$ R‐SO_3_+H_2_O. The latter case served as a catalyst for the production of molecular oxygen.

The longest reaction pathway consisted of steps **i** to **v**. The aforementioned route, **i** to **iii**, was continued by incorporating an OH radical into the molecule in position ortho with respect to the former phenol ring and by adding a second OH radical, resulting in the formation of a cis‐diol. The electronic distribution of structure **v** was verified by computing the Wannier centers, which showed that the radical is located on carbon 2 with the sulfonate ion. The obtained routes are in accordance with the established empirical rules, whereby the most acidic proton is initially abstracted, and subsequent additions are possible according to the directing capacity of the present groups.

### Randomness of the Model

One of the most important factors to consider was the randomness of the model. The water box was automatically generated using the VMD software^[22]^ and the coordinates of the UV filters were inserted into the box, automatically removing the water molecules closer than 1.5 Å to the substrate. The system was then allowed to reach the chemical equilibrium, displacing each atom from its original position. Three sets of eight hydroxyl radicals were created, resulting in a highly oxidizing environment. An alternative to avoid this extreme condition is to create pairs of radicals each time, to wait until both OH are consumed before creating a new pair, and to repeat this several times. Due to the dynamic nature of the system, it would be necessary to determine which hydrogen atoms are part of the solvent, choose between them, and finally decide which product will be the final one. This will lead to a bias if the radicals are placed in specific zones, thus advancing reactions or directing the mechanism towards the formation of desired products, whether intentionally or not. Consequently, it is postulated that the less we intervene during the reactive simulations, the more reliable the model will be.

### Amount of Solvent

Another relevant factor was the number of water molecules to be included. Initially, a system with about 30 solvent molecules was considered to simulate the first solvation layers, but as the dynamics progressed, it was observed that the water molecules quickly moved away from the hydrophobic regions, thus creating holes in the cell. To avoid this, a new system was created that was completely filled with water molecules, approaching a density of 1 g cm^−1^. In these new systems, it was initially possible to maintain a constant water concentration around the entire molecule, but the increased amount of hydrogen bridging quickly connected the radicals to each other, resulting in a higher incidence of side reactions such as the production of H_2_O_2_ or O_2_. As a result, systems containing more solvent generally resulted in shorter pathways, but showed the same reactivity trends that could be extrapolated.

## Conclusions

In this work, a theoretical approach to the electrochemical treatment of water using ab‐initio molecular dynamics simulations was applied to the case of the UV filters BP3 and BP4, analyzing different factors and parameters of the dynamics. The proposed model shows the most reactive zones of each molecule and some probable oxidation routes. Since the experimental treatment will involve a larger time and amount of reactants, the products obtained can be considered as intermediates of longer routes. As for the types of ensembles, no significant difference was observed between them. The NVE assembly was tested using CPMD and BOMD, and in both cases, both short and long reaction pathways were obtained, with the higher computational cost of BOMD being the most significant difference. Comparing the NVE and NVT ensembles, there was no difference in reactivity. A much more relevant factor was the initial position of the radicals, so it is necessary to create different sets of radicals.

Another factor studied was the amount of solvent. Although including more solvent molecules resulted in a more realistic model, fewer solvent molecules tended to have longer oxidation pathways, thus increasing the range of potential products. Since each system is different, an optimal amount of solvent cannot be established, but it should be ensured that there is enough solvent to create at least two solvation layers, to generate at least three sets of radicals, and not to leave large voids in the cell.

With regard to the reactivity of BP3 and BP4, the empiric rules of reactivity were respected in all systems, thereby demonstrating that this model is an appropriate approach to the treatment of these UV filters with an oxidizing environment. The main trends observed were as follows: first, the abstraction of acidic protons; second, the addition of OH radicals to the rings; and third, the oxidation of the functional groups, which can lead to major structural changes as ring closures and cleavages. After the first oxidation in a ring, the second oxidation occurs in ortho or para position. We consider all the products just as intermediates of longer pathways, because according to what has been observed, alcohols can still be oxidized to carbonyls or lead to oxidative cleavages, and both rings can react. The ultimate formation of highly toxic quinones seems likely. Considering the different kinds of reactions observed in the dynamics, it can be established that this approach is sufficiently flexible to model either acid‐base, redox, and radical reactions, given that they have been observed in these dynamics. We reiterate the importance of considering different sets of reactants in different random positions to avoid any model bias.

## Methodology

The methodology used was described previously.[^23^, ^24^, ^25^, ^26^] Firstly, the geometry of each molecule was optimized by DFT calculations, employing the Becke‐Lee‐Yang‐Parr (**BLYP**)[^27^, ^28^] level of theory, a cutoff of 70 Ry and the Troullier‐Martins pseudo‐potentials optimized for BLYP (**MT‐BLYP**).^[29]^ Secondly, the optimized structure was then introduced in a suitable cubic “box of water”. The dynamics were performed using the BLYP method, MT‐BLYP pseudo‐potentials and a cutoff of 70 Ry, a time step of 4 a.u. and a fictitious electron mass of 400 a.u. Hereby, the value for the fictitious electron mass was chosen as the default value, while the time step was decreased from the default value of 5 a.u. in order to guarantee that the fast electron dynamics is well described. The system was let to evolve by itself at a temperature of 300±100 K over 10 000 steps (almost 1 ps), reaching the state of equilibrium. The BLYP functional was chosen as it represents an excellent compromise between cost and accuracy for simulations using large plane‐wave basis sets. Plane‐wave basis sets are strongly superior to Gaussian basis sets when solving the CPMD equations, as they lead to no basis‐set superposition error and to no artificial forces from pulling basis functions through space.[^30^, ^31^]

Thirdly, in order to simulate the oxidizing environment, eight hydroxyl radicals were generated in the simulation cell. The number of eight radicals results from our experience from previous studies: It guarantees high reactivity on the picosecond timescale, but is not too high as to produce side reactions and too strongly oxidative conditions. The dynamics were performed using all the aforementioned parameters. The Local Spin Density (**LSD**) correction must be considered because there are open‐shell species involved. The molecular dynamics runs were restarted until every OH radical had reacted, or the system reached equilibrium. These simulations show feasible oxidative processes that every molecule would follow.

For comparison, the previously described dynamics were repeated using the Nosé‐Hoover thermostat[^32^, ^33^] with a total kinetic energy of 0.01 a.u. and a frequency of 10 000 cm^−1^ for the electrons, and a temperature of 300 K and a frequency of 3 000 cm^−1^ for the ions. The frequency for the ions was chosen as a typical frequency in the system (O−H stretch), to which the thermostat may couple, while the frequency of the lighter and faster electrons was chosen somewhat higher. Thus, it was possible to compare the behavior of the organic UV filters in the NVE and NVT ensembles. These simulations were performed using different amounts of water molecules, to observe the effect of the amount of solvent in the dynamics. BOMD with similar parameters was also tested for BP3. A total of 15 dynamics were run for BP3 and 12 for BP4. The complete scheme with the products and intermediaries can be found in the Supplementary Material, Figures S1 and S3.

The structure of every intermediate and product was optimized using the BLYP, B3LYP^[34]^ and B2PLYP^[35]^ levels of theory, with the basis set 6‐311G(d,p) as implemented in Gaussian 16. The GD3BJ dispersion correction^[36]^ was employed.

Sample movies with Wannier functions and spin densities are provided in the Supplementary Material, together with the corresponding input files for CPMD version 4.3.

## Conflict of Interests

The authors declare no conflict of interest.

1

## Supporting information

As a service to our authors and readers, this journal provides supporting information supplied by the authors. Such materials are peer reviewed and may be re‐organized for online delivery, but are not copy‐edited or typeset. Technical support issues arising from supporting information (other than missing files) should be addressed to the authors.

Supporting Information

## Data Availability

The data that support the findings of this study are available from the corresponding author upon reasonable request.
