# Supplementary material for: Electrochemical Oxidation of UV Filters: A First‐Principles Molecular Dynamics Study
Source: Chemistry. 2024 Nov 16;30(71):e202402924. doi: 10.1002/chem.202402924 (PMC11653232; doi:10.1002/chem.202402924)
Supplement: Supplementary file 1 — Supporting Information [file CHEM-30-e202402924-s001.pdf]

Chemistry–A European Journal

Supporting Information

Electrochemical Oxidation of UV Filters: A First-Principles Molecular Dynamics Study

Luis Álvarez and Irmgard Frank*

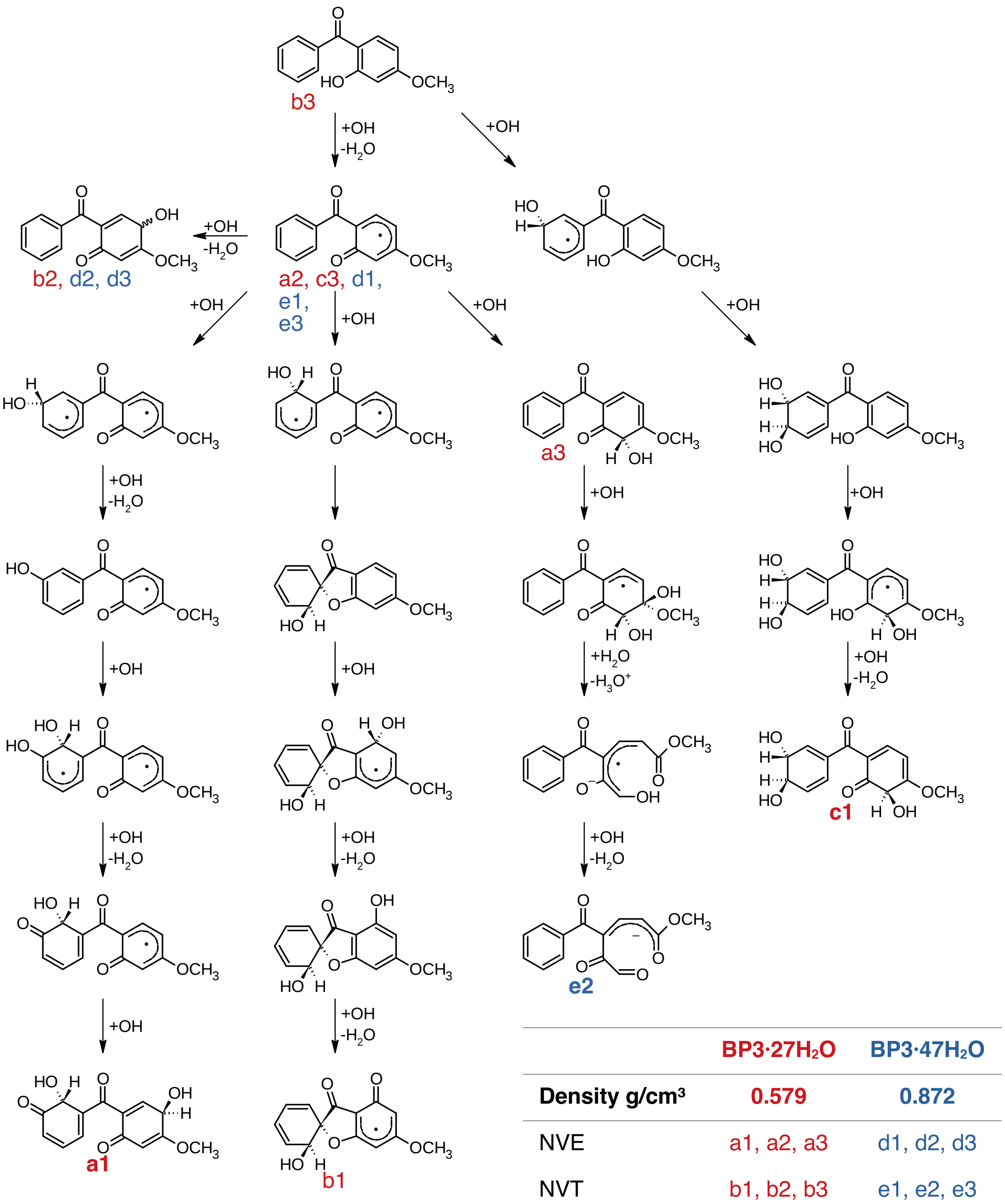

	BP3·27H₂O	BP3·47H₂O
**Density g/cm³**	**0.579**	**0.872**
NVE	a1, a2, a3	d1, d2, d3
NVT	b1, b2, b3	e1, e2, e3
BO-NVE	c1, c2, c3	–

Figure S1: Complete scheme of the oxidation pathways followed by **BP3**. The indexes represent the final structure of each dynamics run, whose parameters are shown in the table. Routes a1, c1 and e2 are explained in detail in the **Figure 1** as **a**, **b** and **c**.

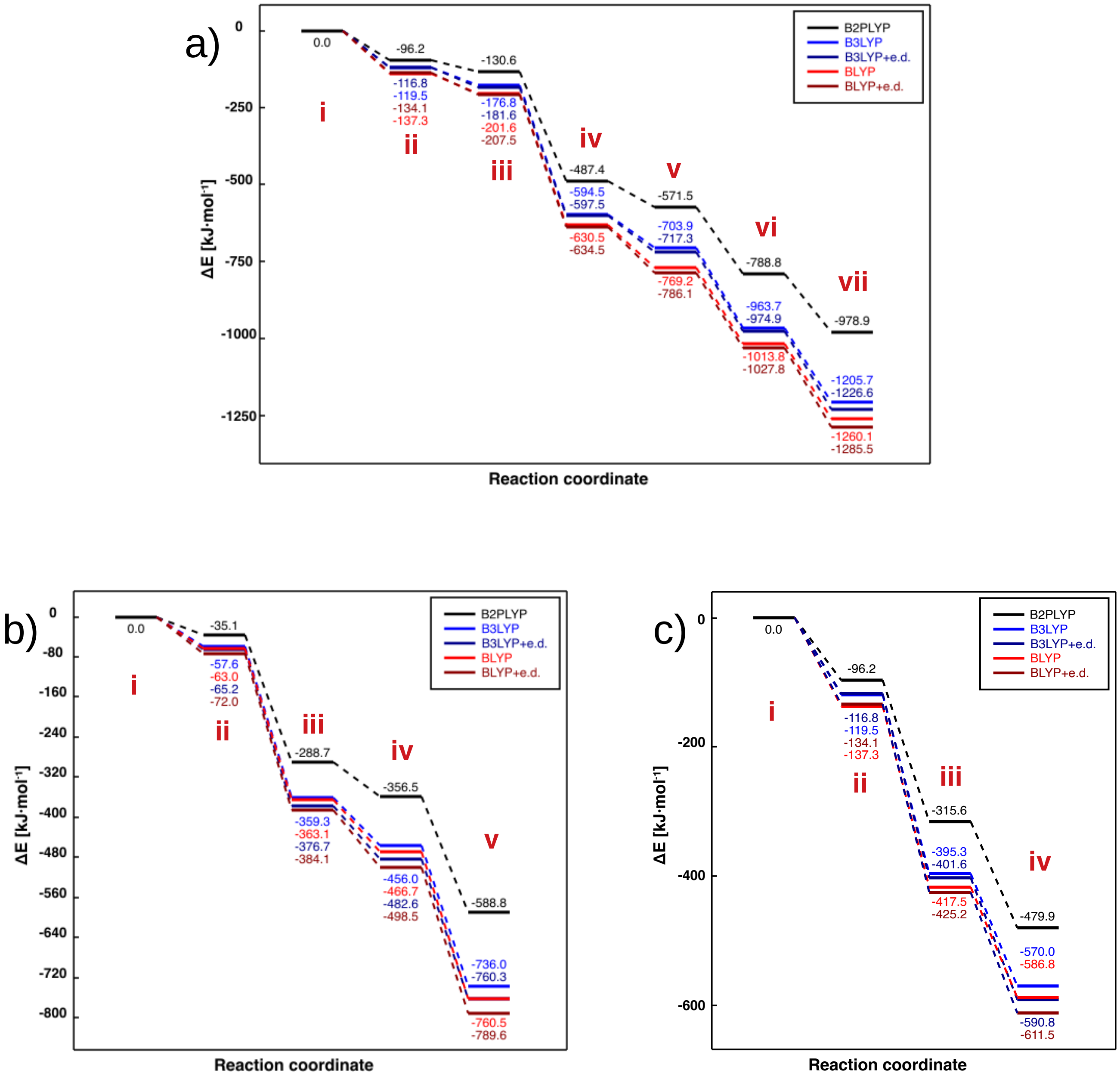

Figure S2: Reaction energy of reaction pathways a1, c1 and e2 (see **Figure S1**). The letters **a**, **b** and **c** and numbering corresponds with the routes and intermediates from the main document, **Figure 1**.

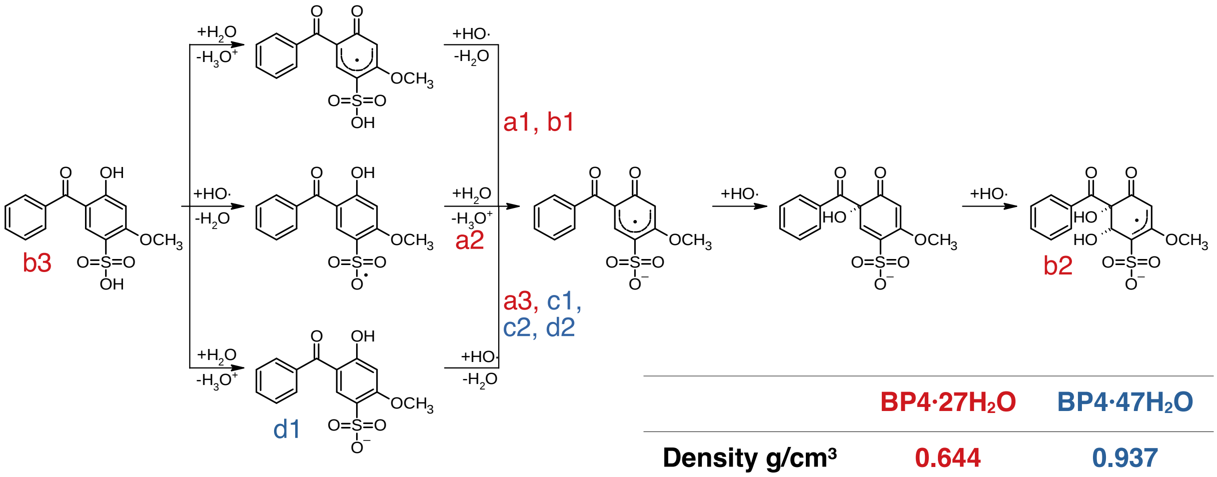

	BP4·27H$_2$O	BP4·47H$_2$O
**Density g/cm^3**	**0.644**	**0.937**
NVE	a1, a2, a3	c1, c2, c3
NVT	b1, b2, b3	d1, d2, d3

Figure S3: Complete scheme of the oxidation pathways followed by **BP4**. The indexes represent the final structure of each dynamics run, whose parameters are shown in the table.
